# Personalized Ultra-Fractionated Stereotactic Adaptive Radiotherapy (PULSAR) for Patients with Lung Tumors and Severe Pulmonary Disease

**DOI:** 10.3390/jcm15031261

**Published:** 2026-02-05

**Authors:** Kenneth D. Westover, Ruiqi Li, Stetler Tanner, Maureen Aliru, Mu-Han Lin, Bin Cai, David Parsons, Justin Visak, Yesenia Gonzalez, Anundip Gill, Yuanyuan Zhang, Shahed N. Badiyan, Puneeth Iyengar, Robert Timmerman

**Affiliations:** 1 Department of Radiation Oncology, The University of Texas Southwestern Medical Center at Dallas, Dallas, TX 75390, USA; 2 Department of Biochemistry, The University of Texas Southwestern Medical Center at Dallas, Dallas, TX 75390, USA; 3 MSKCC—Memorial Sloan Kettering Cancer Center, New York, NY 10065, USA

**Keywords:** SABR (stereotactic ablative radiotherapy), SBRT (stereotactic body radiation therapy), PULSAR (personalized ultra-fractionated stereotactic adaptive radiotherapy), ILD (interstitial lung disease), COPD (chronic obstructive pulmonary disease), lung cancer

## Abstract

**Background/Objectives:** Patients with early-stage non-small cell lung cancer (NSCLC) or limited lung metastases and compromised lung function, such as those with interstitial lung disease (ILD) or chronic obstructive pulmonary disease (COPD), or other factors rendering them high-risk for surgery or medically inoperable, face increased risks of treatment-related toxicity from stereotactic ablative radiation therapy (SABR). This study evaluated a novel treatment approach to mitigate these risks. **Methods:** We investigated Personalized Ultra-Fractionated Stereotactic Adaptive Radiotherapy (PULSAR), delivered as pulsed radiation every three weeks, in patients with <5 cm lung tumors and ILD, COPD, or prior therapy. Treatment occurred between 2022 and 2024. Online adaptive radiotherapy (o-ART) was employed in 20 patients (80%) to modify treatment plans when anatomical changes warranted replanning. Primary outcomes included volumetric tumor response, changes in dose to organs at risk (OARs) and acute events, while secondary outcomes included local and tumor control, and overall survival. **Results:** Twenty-three patients received PULSAR treatment at doses between 40 Gy and 60 Gy in 5 fractions and one patient received 54 Gy in 3 fractions, with a median follow-up time of 16.2 months. Approximately half of treated patients demonstrated volumetric tumor response, with median residual volume of 70% (range 36–100%) at maximal response. Among the 20 patients (80%) who underwent online adaptive replanning, significant reductions in OAR dosimetry were observed for all organs assessed including the Dmax for heart (*p* = 0.0053), bronchus (*p* = 0.0003), esophagus (*p* = 0.0005), spinal cord (*p* = 0.025), and the lung V20 Gy and V12.5 Gy (*p* < 0.0001). Treatment-related toxicity included two grade 1–2 adverse events and six grade 3 events consisting of pneumonitis, dyspnea or lung infection, with no grade 4 or 5 events. Median progression-free survival was 21.1 months, with 1-year overall survival of 74% and 1-year local control of 100%. **Conclusions:** PULSAR shows promise as a feasible treatment option for high-risk patients with NSCLC or lung metastases, demonstrating no grade 5 events and complete tumor control. Additional research is needed to fully evaluate the safety profile of PULSAR in the high-risk subgroups and whether PULSAR’s treatment intervals and adaptive planning advantages lead to improved long-term outcomes compared to conventional, uninterrupted SABR regimens.

## 1. Introduction

Stereotactic Ablative Radiation (SABR), also known as Stereotactic Body Radiotherapy (SBRT), was originally developed as a treatment for patients with lung cancer who are not candidates for surgical resection due to poor pulmonary function, advanced age, or other comorbidities. Multiple trials have demonstrated that SABR achieves high local control rates of approximately 90–95% at 3 to 5 years of follow-up [1,2,3,4]. However, these trials also reported grade 3 or higher toxicity rates ranging from 15 to 30%, with certain factors—such as centrally located tumors, associated with higher toxicity rates [5]. Patients with interstitial lung disease (ILD) or severe chronic obstructive pulmonary disease (COPD) face an even greater risk of high-grade toxicity, likely attributable to their compromised baseline pulmonary reserve and the inherent fragility of diseased lung parenchyma [6,7]. Most studies report significant rates of high-grade toxicity in these populations. COPD patients may experience acute exacerbations, prolonged respiratory decompensation, and decreased quality of life following treatment [8]. Historical data suggest grade 3 or higher toxicity rates of 10–20% in this population, with some studies reporting treatment-related mortality. For ILD, alarmingly, case series have documented SABR-related death rates as high as 60% in patients [9]. Nonetheless, a recent prospective study of 39 ILD patients treated with 50 Gy in 5 fractions demonstrated a SABR-related death rate of 7.7% [7]. The balance between oncologic benefit and treatment-related morbidity in these patients with already limited pulmonary reserve necessitates novel treatment approaches that preserve tumor control while minimizing toxicity risk [10,11,12].

To address these high-risk populations, we hypothesized that increasing the time interval between SABR fractions could improve outcomes. This strategy, termed Personalized Ultra-Fractionated Stereotactic Adaptive Radiotherapy (PULSAR) (Figure S1) [13], leverages extended inter-fraction intervals to permit recovery of normal tissues from acute inflammatory injury and to potentially mitigate progressive fibrotic responses. Additionally, tumor shrinkage, typically observed approximately three weeks after the start of the therapy [14], could retract tumors away from normal tissues, reducing toxicity when treatment plans are re-optimized at each radiation pulse. Furthermore, PULSAR has been observed to induce anti-tumor immune responses in preclinical models [13,15]. Delivering radiation at three-week intervals may enhance these immune responses, potentially increasing treatment tolerance without compromising tumor control.

This study evaluated the use of PULSAR, with radiation pulses delivered every three weeks, in patients with thoracic cancers deemed high risk for traditional SABR regimens. Online adaptive radiotherapy (o-ART) facilitated this approach for the majority of cases, enabling real-time modifications to radiation treatment plans in response to anticipated anatomical changes. This study presents our early experience with PULSAR in lung cancer treatment, focusing on tumor changes during therapy, short-term treatment-related toxicity, and the impact of o-ART on normal tissue sparing.

## 2. Materials and Methods

### 2.1. Study Design

This was a single-institution retrospective analysis of consecutively treated patients. Patients were treated between November 2022 and January 2024. We identified patients with early stage (T1-T2N0) NSCLC (*n* = 22) or limited lung metastases (*n* = 2) who were judged to be medically inoperable by a thoracic surgeon. Most patients were treated with doses of 40 Gy, 50 Gy, or 60 Gy in 5 fractions with each fraction given every 3 weeks; one patient received 54 Gy in 3 fractions. Patients were followed with serial computed tomography (CT) scans and office visits every 3 months during the follow-up period.

### 2.2. Participants

Patients were required to have a diagnosis of T1-T2N0 NSCLC or limited lung metastases in the setting of ILD, severe COPD (defined as GOLD stage 3–4, FEV1 < 50% predicted, or clinical assessment of high pulmonary risk), or prior thoracic irradiation necessitating re-treatment. Patients were deemed inappropriate for surgical resection based on thoracic surgical consultation. Empiric treatment was permitted for patients deemed high risk for biopsy following multidisciplinary tumor board discussion and radiographic evaluation consistent with malignancy per national guidelines. Eastern Cooperative Oncology Group (ECOG) performance status ranged from 0 to 3.

### 2.3. Procedures

Patients were immobilized using a rigid body frame system to ensure reproducible positioning. Prior to treatment, a planned CT scan was performed with the patient in the body frame for image registration and target localization. All patients were treated under free-breathing conditions. For tumors demonstrating >10 mm motion in any direction on 4-dimensional CT simulation, abdominal compression was applied to reduce respiratory excursion. Deep inspiration breath-hold technique was not employed given the severely compromised pulmonary function in this high-risk cohort. The internal target volume (ITV) was created by encompassing all positions of the gross tumor volume across all respiratory phases. A planning target volume (PTV) was generated by applying a uniform 5 mm expansion to the ITV to account for setup uncertainty. Target coverage objectives required that 95% of the PTV receive at least 100% of the prescription dose. Online adaptive radiotherapy was performed at the treating physician’s discretion based on visible anatomical changes on setup cone-beam CT or MRI imaging. Optimal imaging parameters for each adaptive fraction were selected based on the physician’s preference. When employed, contours were re-delineated and plans re-optimized to account for tumor regression and anatomical changes while maintaining identical dose constraints for organs at risk [16].

### 2.4. Outcomes

The primary end point was short-term (6-month) treatment-related toxicity. Secondary outcomes included tumor changes during treatment and follow-up, improvements in OAR metrics, cancer control, and overall survival. Radiographic assessment during follow-up was performed with chest CT every 3 months. Tumor recurrence was defined as radiographic or pathologic evidence of cancer progression leading to additional therapy or tumor-related death.

### 2.5. Statistical Analysis

Kaplan–Meier analysis was performed to evaluate distant metastasis-free survival and overall patient survival; cumulative incidence analysis was performed for local control to account for death as a competing risk. Comparisons were made using the log-rank test for survival outcomes. No adjustment for multiple comparisons was applied given the exploratory nature of this retrospective study. All three survival endpoints were considered secondary outcomes of equal interest. Organ-at-risk (OAR) dose metrics for initial versus adaptive treatment plans at maximal tumor response were compared using Wilcoxon signed-rank test for paired data. Statistical significance was set at *p* < 0.05. All analyses were conducted using Prism Version 10.5.0 (GraphPad Software, San Diego, CA, USA) and R Studio Version 2025.09.1 (Posit PBC, Boston, MA, USA).

## 3. Results

### 3.1. Patient Characteristics

The study included 24 patients with a total of 25 tumors (Table 1) with a median age of 72 years (interquartile range [IQR]: 65–82), with 67% being female. Sixty-eight percent of patients had T1 tumors, while 32% had T2 tumors, based on size criteria. The median baseline primary tumor size was 25 mm (IQR: 17–33). Of these, 36% were in a central location, defined as being within 2 cm of the proximal bronchial tree or adjacent to the heart. Performance status, as assessed by the Eastern Cooperative Oncology Group (ECOG) scale, showed that 50% of patients had an ECOG score of zero or one, 38% had a score of two, and 13% had a score of three. The primary indications for PULSAR treatment were ILD (29%), COPD (67%), and re-irradiation (8%). Among those with available data, 67% had a history of smoking, with a median smoking history of 31 pack-years (IQR: 15–39). Baseline lung function tests, for those patients tested, showed a median forced expiratory volume in 1 s (FEV1) of 62% of predicted normal (IQR: 43–86), and a median diffusing capacity (DLCO) of 33% of predicted normal (IQR: 22–39). Treatment with PULSAR due to COPD did not have strict GOLD classification criteria; this was based on clinical judgment related to functional limitations due to pulmonary symptoms.

### 3.2. Treatment Characteristics

Treatments occurred between November 2022 and January 2024. Most tumors were treated with five fractions of PULSAR, with 42% receiving 60 Gy, 31% receiving 50 Gy, and 19% receiving 40 Gy (Table 2). One patient received 54 Gy in three fractions. Tumor laterality was nearly balanced, with 54% of tumors on the left side and 42% on the right. CT-adaptive radiation therapy was used in 77% of cases, MRI-adaptive techniques in 8%, and non-adaptive techniques in 15%. The median treatment duration was 83 days (range 52–103 days).

### 3.3. Changes in Tumor Volumes

**Figure 1:**
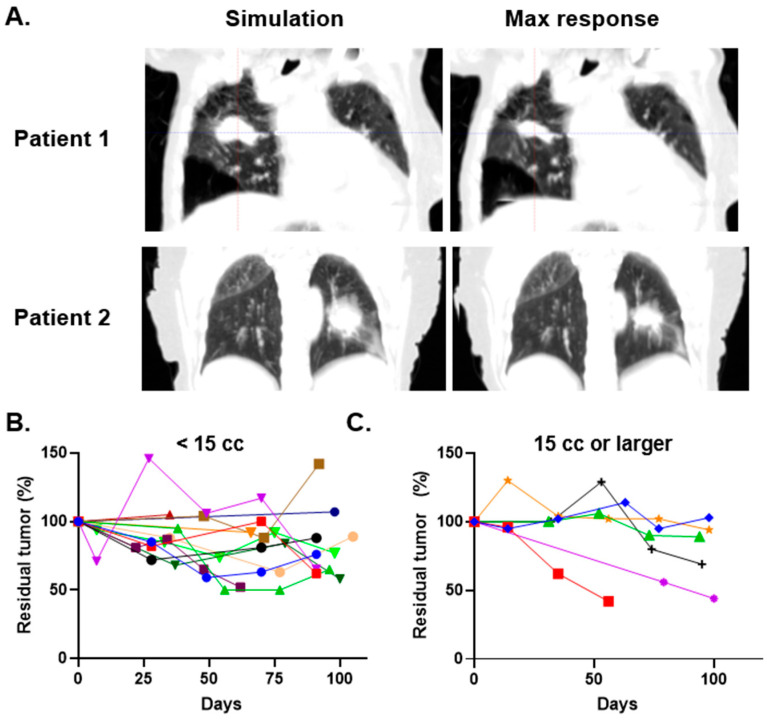
Tumor shrinkage during PULSAR treatment. (**A**) Representative axial computed tomography images at simulation (**left**) and maximal tumor response (**right**) for two patients demonstrating substantial volumetric reduction. Patient 1 (**upper panels**) and Patient 2 (**lower panels**) show regression of lung tumors between treatment fractions. (**B**) Longitudinal tumor volume measurements expressed as percentage of initial internal target volume for tumors < 15 cc at simulation (*n* = 11), with each colored line representing an individual patient. Measurements were performed at simulation (day 0) and at each subsequent fraction. (**C**) Longitudinal tumor volume measurements for tumors ≥ 15 cc at simulation (*n* = 13). The timing and magnitude of tumor regression varied considerably among patients.

For patients treated with adaptive therapy, we evaluated tumor changes over time using tumor segmentation volumes from o-ART plans. Tumor volumes were obtained directly from the o-ART treatment planning system using ITVs defined by physicians at the time of treatment. Median residual tumor volume at maximal response was 70% of initial volume (IQR 54–88%). The strongest responders showed a decrease in the internal target volume (ITV) to less than 50% of the initial volume in four cases (Figure 1). There appeared to be an association between large initial tumor size and tumor shrinkage during treatment, though variability was observed even within subgroups of tumors divided by size (Figure 1B,C and Figure S2). The timing of maximal tumor shrinkage varied considerably, occurring at fraction 2 in six patients, fraction 3 in eight patients, fraction 4 in four patients, and fraction 5 in two patients. No consistent pattern emerged correlating timing of response with tumor size, location, or prescribed dose. These findings demonstrate heterogeneous volumetric responses to PULSAR among NSCLC tumors, with larger initial tumor volumes associated with greater absolute and proportional shrinkage, though substantial regression was also observed in some smaller tumors.

### 3.4. Changes in Dosimetry

**Figure 2:**
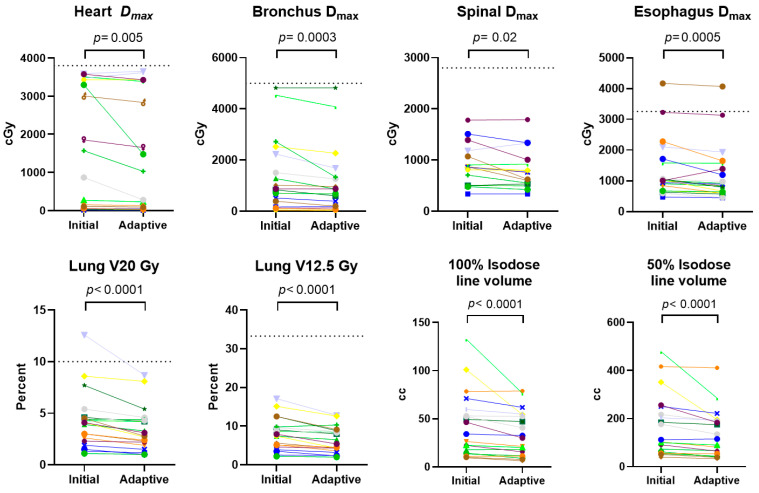
Organ-at-risk dosimetric comparison between initial and adaptive treatment plans. Paired analysis of organ-at-risk dose metrics for 20 patients who underwent online adaptive replanning. Each colored line represents an individual patient, connecting the initial simulation plan value (left) to the adaptive plan value at maximal tumor response (right). Dotted horizontal lines represent institutional organ-at-risk constraint goals. Statistical comparisons were performed using paired analysis.

Given the observed tumor shrinkage, we hypothesized that dosimetry to organs at risk would improve correspondingly. A comparative analysis of dosimetric and volumetric parameters in the 20 patients who underwent adaptive treatment (Figure 2, Table S1) showed significant reductions in organ-at-risk (OAR) doses when comparing the initial radiation plans to adaptive plans corresponding to the minimal tumor volume observed (maximal tumor response) during the adaptive course. For the heart, a median reduction in maximum dose (Dmax) of 19.0 cGy was observed with the adaptive plan (*p* = 0.0053). Significant reductions in Dmax were also observed for the bronchus (median reduction 87.4 cGy, *p* = 0.0003), esophagus (median reduction 91.3 cGy, *p* = 0.0005), and spinal cord (median reduction 58.5 cGy, *p* = 0.025). Lung V20 Gy and V12.5 Gy both showed significant reductions with the adaptive plan (median reductions of 0.6% and 1.0%, respectively, both *p* < 0.0001). In one case, the Lung V20 Gy was higher than the clinically acceptable threshold at the start of treatment but became acceptable with adaptive treatment. Significant reductions in the volumes enclosed by the 100% and 50% isodose lines were also observed (median reductions of 3.1% and 12.4% respectively, both *p* < 0.0001). Although few of these reductions led to converting OAR metrics from clinically unacceptable to acceptable thresholds, these results demonstrate the potential benefit of adaptive PULSAR in reducing radiation exposure to critical structures.

### 3.5. Adverse Events

Toxicity was assessed with CTCAE v5.0 grading at each treatment visit and at each follow-up visit. Within the first six months following treatment completion, we observed three grade 1–2 adverse events (fatigue in two patients, mild cough in one patient) and six grade 3 events. Grade 3 events included dyspnea in four patients, pneumonitis in one patient, and lung infection in one patient (Table S2). We note that three of these events happened in our ILD subgroup of patients. One patient involved an 83-year-old woman with a pre-treatment DLCO of 25% of predicted. She was admitted for what was felt to be a COPD exacerbation 3 months after completion of treatment. Another was an 85-year-old man with a pre-treatment DLCO of 34% of predicted who was admitted for hypoxic respiratory failure 3 months after the completion of treatment. Prior to PULSAR treatment, this patient was told by his pulmonologist that he had 2–3 weeks life expectancy because of the pulmonary disease. The last was a 69-year-old with pretreatment DLCO of 17% of predicted who increased oxygen usage due to dyspnea 4 months after treatment. Notably, no grade 4 or 5 events were observed within the 6-month post-treatment window. All adverse events were considered treatment related. These findings suggest that PULSAR is associated with low rates of grade 4–5 toxicity in patients with ILD or severe pulmonary disease, although one in four patients experienced a grade 3 event.

### 3.6. Cancer Outcomes

**Figure 3:**
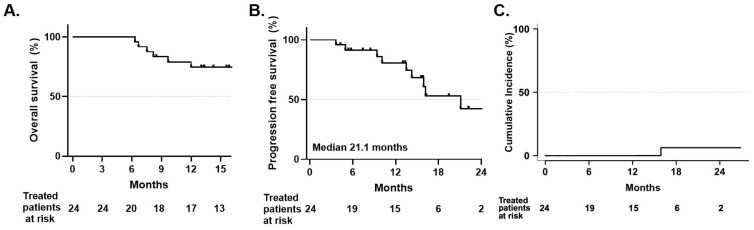
Cancer outcomes. (**A**) Overall survival estimated by Kaplan–Meier method, with a 1-year overall survival of 74%. Median overall survival was not reached during the follow-up period (median 16.2 months). Numbers at risk shown below *x*-axis. (**B**) Progression-free survival estimated by Kaplan–Meier method. Median PFS was 21.1 months (95% CI: 13.5 months—not reached), with 1-year progression-free survival of 80%. Numbers at risk shown below *x*-axis. (**C**) Cumulative incidence of local recurrence calculated using competing risk analysis, with death from any cause treated as a competing event. The 1-year cumulative incidence of local recurrence was 0%.

Since PULSAR was delivered at 3-week intervals over a median treatment course of 85 days, concerns about potential tumor repopulation or systemic disease progression during therapy were considered. With a median follow-up of 16.2 months (range 4.3–35.1 months), there were no cases of local relapse, indicating that local progression is uncommon in the initial post-treatment phase (Figure 3A). Systemic progression was the most common mode of cancer progression occurring in six patients. One patient did not finish their PULSAR course because of systemic progression. Median progression-free survival was not reached, and overall survival was 74% at 1 year (Figure 3B,C). This survival rate compares favorably to historical data for patients with severe COPD treated with SBRT, where 1-year survival rates are typically 50–60% [17].

## 4. Discussion

For patients with early-stage NSCLC or limited lung metastases and severely compromised lung function, treatment decisions are frequently determined by the risks associated with therapy, which can lead to recommendations for active surveillance or best supportive care. In this study, we demonstrate that thoracic PULSAR, administered at a 3-week interval, is feasible and may provide a viable alternative to non-treatment or other high-risk treatments. A significant proportion of patients treated with PULSAR experienced substantial tumor size reductions and corresponding decrease in radiation doses to normal tissues. The rate of serious acute toxicities was also numerically lower than observed in previous studies.

Interpretation of findings related to ILD patients, particularly toxicity, warrants careful consideration. While the recently reported ASPIRE-ILD trial [7], a prospective phase 2 study of 50 Gy in 5 fractions delivered over 2 weeks in 39 ILD patients provides a relevant benchmark for ILD patients treated with SABR, direct comparison with our results is complicated by several important differences. First, our ILD subgroup was small consisting of seven patients, making statistical comparisons challenging to interpret. Second, we note that the severity of pulmonary disease may have been more severe in our cohort based on the median DLCO of 33% in our study (in the 16 patients where it was measured), compared to a median of 49% in the ASPIRE-ILD study. The ASPIRE-ILD trial reported 1-year overall survival of 79%, grade 3 or higher toxicity of 18% (including 8% grade 5), and 2-year local control of 92%. Our ILD subgroup demonstrated 1-year overall survival of 50%, grade 3 events in 3 out of 7 (43%) patients, and 100% local control at 1 year. The difference in baseline pulmonary function between the two studies could account for the higher rate of grade 3 events and lower overall survival observed in our ILD subgroup compared to ASPIRE-ILD. Second, these studies had important methodological differences. In our study, we focused on acute events (6 months), considering that events after that time would be difficult to assign to radiation vs. the natural progression of pulmonary disease. However, in ASPIRE-ILD, all pulmonary related deaths were considered treatment related, and the timing was not specified. Untreated early-stage NSCLC in ILD patients has median survival of 6–12 months, and many studies have demonstrated high competing mortality from ILD progression itself with rates in the literature approaching 30–50% at 2 years [18,19]. Regardless of these caveats related to cross-study comparisons and competing risks from the natural history of ILD, we note that our 43% rate of grade 3 toxicity is high and acknowledge that some proportion of this is likely related to radiation treatment.

These observations raise the possibility that stratifying patients by baseline pulmonary function may be important for interpreting outcomes in future studies of PULSAR and other radiation approaches in high-risk populations. DLCO has been shown to be a prognostic marker in both ILD and COPD. In ILD, the GAP (Gender, Age, Physiology) staging system incorporates DLCO as a variable for predicting mortality [20], and studies have reported that patients with DLCO below 35% of predicted have worse survival [21]. Moreover, in COPD, recent investigations have shown that long-term survival following hospitalization appears to correlate with disease severity, with DLCO serving as an independent prognostic factor [22]. If baseline pulmonary function is indeed a major determinant of outcomes in these patients, prospective stratification by DLCO may help distinguish the contribution of radiation-related toxicity from the expected natural history of progressive pulmonary disease. Standardized reporting of baseline pulmonary function metrics, particularly DLCO, may therefore be valuable for future trials enrolling patients with compromised lung function.

Our study suggests that reductions in OAR doses achieved through adaptive planning contributes, or has the potential to contribute, to lower radiation-related toxicity in high-risk individuals. ART was used to respond to tumor shrinkage and anatomical changes, improving dosimetry throughout the treatment course. While the majority of patients met institutional OAR constraint goals at the outset of treatment, adaptive replanning resulted in further dose reductions in most cases. Importantly, for the few patients who initially exceeded institutional dose constraints, adaptive planning provided the opportunity to bring these metrics into acceptable ranges as tumors regressed during treatment. This feature of PULSAR may be particularly valuable for patients with large or centrally located tumors where initial treatment plans approach or exceed OAR tolerance doses. Future advancements could integrate radiomic features or biological markers, such as blood-based indicators, to guide treatment decisions like radiation dose modifications or the addition of systemic therapies. If favorable results remain after longer follow-up, PULSAR may also be extended to lower-risk patients, where its personalization benefits could be even more impactful.

A key observation was the high rate of local disease control. While conclusions about long-term control cannot be drawn yet, these findings suggest that PULSAR does not compromise tumor control. This is significant because extending the interval between treatment sessions could theoretically enable tumor repopulation. However, the relevance of this concept to hypofractionated schemes is uncertain, as individual fraction doses in the ablative range (8–12 Gy) exceed the shoulder region of classical dose–response survival curves, rendering linear-quadratic modeling less reliable [23]. Additionally, PULSAR may elicit immune responses that sustain tumor control between radiation pulses [24]. While theoretical, if true, this could preserve the high local control rates associated with traditional SABR while enhancing systemic control over time. In patients with fewer competing risks, this could offer significant advantages for PULSAR over standard SABR and raises the intriguing hypothesis that combining PULSAR with immunotherapy might yield synergistic benefits.

The extended treatment duration inherent to PULSAR also introduces a methodological challenge when comparing outcomes to conventional SABR. Standard SABR courses are typically completed within one to two weeks, whereas our PULSAR regimen spanned a median of 83 days. This difference complicates the assessment of both cancer outcomes and treatment-related toxicity, which are traditionally measured from treatment completion. For a patient completing PULSAR, disease progression or toxicity occurring at “3 months post-treatment” actually occurs approximately 6 months from treatment initiation, compared to approximately 3.5 months from initiation for standard SABR. This temporal discrepancy raises the question of whether treatment start date might serve as a more appropriate anchor for outcome measurement when comparing these regimens. The biological rationale of such an approach is supported by the fact that radiation-induced injury begins at first exposure, regardless of when the final fraction is delivered. Future comparative studies of PULSAR versus conventional SABR should prespecify whether outcomes will be measured from treatment initiation or completion, and investigators should consider reporting both timepoints to facilitate meaningful cross-study comparisons.

A key parameter of PULSAR therapy that will require further study and optimization is radiation dosing. In our study, all but one patient received a 5-fraction regimen, with radiation “pulses” ranging from 8 to 12 Gy. Immune responses likely depend on the generation of inflammation in the tumor microenvironment, which may not occur with lower, non-ablative doses. However, radiosensitive tumors, such as small cell lung cancer, even low-dose PULSAR might be effective. Further studies will be needed to explore the full potential and applications of PULSAR therapy.

Although this study focused on patients with ILD and severe COPD, PULSAR may also be applicable to other high-risk groups, such as those with large or central tumors, patients who have undergone prior radiation to a recurrence area, or those treated with anti-angiogenic agents (AAA). We note that approximately a third of patients in this study had central tumors. For recurrent cancer, SABR re-irradiation has demonstrated favorable local control, with approximately 50% local control at 3 years and low rates of grade 3 or higher toxicities in selected patients [25]. Similarly, SABR has been associated with high rates of hemoptysis in patients receiving AAAs; in one study, 22% of such patients experienced grade 3 or higher toxicities [26]. PULSAR may mitigate these toxicity challenges.

This study has several important limitations. First, the retrospective single-arm design precludes definitive conclusions about the comparative safety of PULSAR versus standard SABR, as differences in toxicity could reflect patient selection, dose prescriptions, or other unmeasured confounders. The patient population was heterogeneous, including ILD, COPD, and re-irradiation patients with varying risk profiles, making subgroup analyses underpowered. Second, the median follow-up of 16.2 months is relatively short for assessing long-term local control and late toxicity after hypofractionated radiation therapy, where some series report late failures occurring beyond 2 years. Longer follow-up is essential to determine the durability of local control. Third, treatment was delivered at physician discretion rather than following a prospective protocol, with variable dose prescriptions and inconsistent use of adaptive planning (80% of patients). This reflects real-world practice but introduces heterogeneity. Fourth, longitudinal pulmonary function testing was not systematically performed, limiting assessment of treatment impact on respiratory function. Finally, the proposed immunologic mechanisms remain speculative, as no translational endpoints (e.g., peripheral immune cell phenotyping, cytokine profiles, circulating tumor DNA) were assessed. Prospective studies incorporating such correlative analyses will be essential to validate these hypothesized mechanisms.

## 5. Conclusions

This single-institution retrospective study demonstrates that PULSAR, delivered at three-week intervals with online adaptive replanning, is a feasible treatment approach for patients with early-stage NSCLC or limited lung metastases who are at high risk for conventional SABR due to severe ILD, COPD, or prior thoracic irradiation. We observed tumor shrinkage in a substantial proportion of patients, enabling adaptive plan optimization that significantly reduced radiation doses to organs at risk. Local tumor control was achieved in all patients at one year of follow up. While grade 3 toxicity occurred in approximately one-quarter of patients, no grade 4 or 5 events were observed within six months of treatment completion. These findings are hypothesis-generating and support further prospective evaluation of PULSAR in well-defined high-risk patient populations, with particular attention to the ILD subgroup where toxicity rates warrant careful monitoring. Comparative studies against conventional SABR regimens will be essential to determine whether the extended inter-fraction intervals and adaptive planning advantages of PULSAR translate into improved long-term outcomes for these challenging patients.

## Figures and Tables

**Table 1:** Baseline Patient Characteristics.

Characteristic	Treated Patients (*N* = 24) [Data Available, No.]
Age (years), median (IQR)	72 (65–82), [24]
Sex, No. (%)	[24]
Male	8 (33)
Female	16 (67)
T stage, No. (%)	[25]
T1	17 (68)
T2	8 (32)
Baseline tumor diameter (mm), median (IQR)	25 (17–33) [25]
ITV volume (cc), median (IQR)	15 (3–21) [25]
Tumor location, No. (%)	[25]
Central	9 (36)
Peripheral	16 (64)
Baseline ECOG, No. (%)	[24]
0	1 (4)
1	11 (46)
2	9 (38)
3	3 (13)
Reason for PULSAR, No. (%)	[24]
ILD	7 (29)
COPD	16 (67)
Retreatment	2 (8)
Smoking history, No. (%)	18 (72) [21]
Smoking pack-years, median (IQR)	31 (15–39) [16]
Baseline FEV1 [% predicted], median (IQR)	62 (43–86) [19]
Baseline DLCO [% predicted], median (IQR)	33 (22–39) [16]

IQR = Interquartile Range.

**Table 2:** Treatment Parameters.

Characteristic	Treated Tumors (*N* = 25) [Data Available, No.]
Treatment Regimens	[25]
40 Gy in 5 fractions	5 (19)
50 Gy in 5 fractions	8 (31)
60 Gy in 5 fractions	11 (42)
54 Gy in 3 fractions	1 (4)
Laterality	[25]
Right	11 (42)
Left	14 (54)
Delivery technique	[25]
Ethos	20 (77)
MRI Linac	2 (8)
Non-adaptive	4 (15)
Duration of treatment (days)	83 (52–103) [25]

## Data Availability

Data will be made available upon reasonable request.

## Supplementary Materials

The following supporting information can be downloaded at https://www.mdpi.com/article/10.3390/jcm15031261/s1, Figure S1: Schematic comparison of SABR and PULSAR; Figure S2: Relationship between tumor volume and response; Table S1: Changes in Dosimetry; Table S2: Adverse Events.
