# Supplementary material for: Personalized Ultra-Fractionated Stereotactic Adaptive Radiotherapy (PULSAR) for Patients with Lung Tumors and Severe Pulmonary Disease
Source: J Clin Med. 2026 Feb 5;15(3):1261. doi: 10.3390/jcm15031261 (PMC12898070; doi:10.3390/jcm15031261)
Supplement: Supplementary file 1 [file jcm-15-01261-s001.zip › jcm-4123824-supplementary.pdf]

SUPPLEMENTARY INFORMATION

Safety and Feasibility of Personalized Ultra-Fractionated Stereotactic Adaptive Radiotherapy (PULSAR) for Lung Tumors and Severe Pulmonary Disease

Authors:
Kenneth D. Westover[1,2], Ruiqi Li[1], Maureen Aliru[1], Mu-Han Lin[1], Bin Cai[1], David Parsons[1], Justin Visak[2], Yesenia Gonzalez[1], Anu Gill[1], Stetler Tanner[1], Yuanyuan Zhang[1], Shahed Badiyan[1], Puneeth Iyengar[3], Robert Timmerman[1]
Affiliations:
[1]Department of Radiation Oncology, The University of Texas Southwestern Medical Center at Dallas, Dallas, Texas, 75390, USA.
[2]Department of Biochemistry, The University of Texas Southwestern Medical Center at Dallas, Dallas, Texas, 75390, USA.
[3]MSKCC

Contact Info:
Correspondence may be addressed to:
Kenneth D. Westover (Kenneth.westover@utsouthwestern.edu)

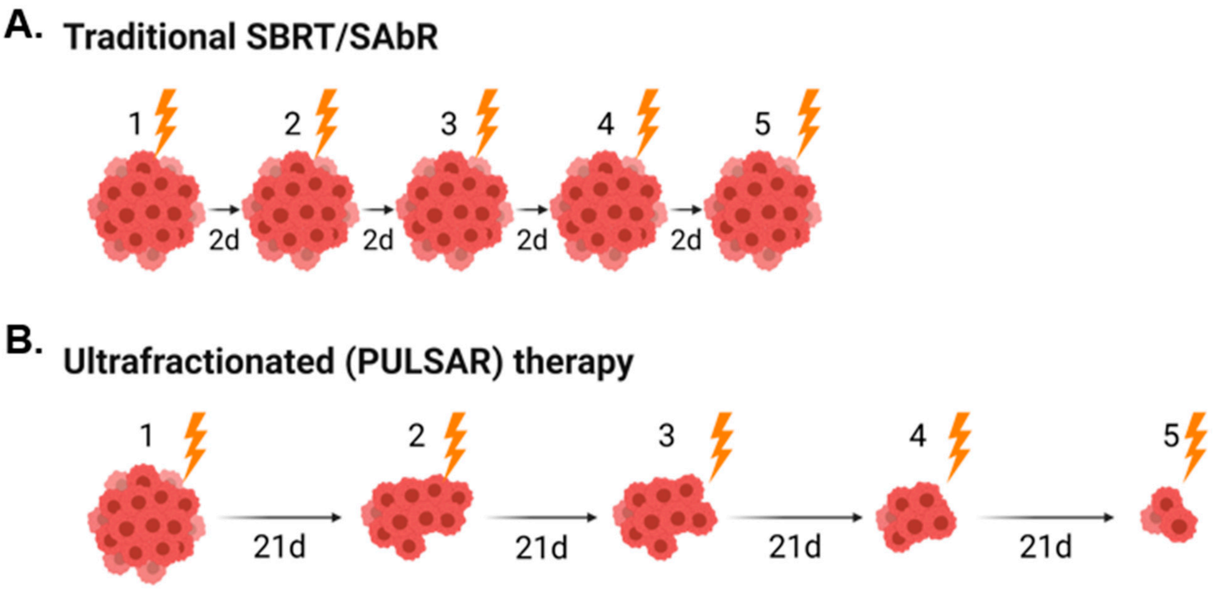

Figure S1. **Schematic comparison of traditional SABR and PULSAR treatment approaches.** *(A) Traditional stereotactic ablative radiotherapy (SABR/SBRT) delivers five fractions on consecutive or alternate days with 2-day intervals, completed within 10-14 days. (B) Personalized Ultra-fractionated Stereotactic Adaptive Radiotherapy (PULSAR) delivers fractions at 21-day intervals, extending treatment to approximately 84 days for a 5-fraction regimen. This prolonged interval allows time for tumor regression and normal tissue recovery between treatments, with the opportunity for adaptive replanning at each fraction. Lightning bolt symbols represent individual radiation fractions; red spheres represent tumors.*

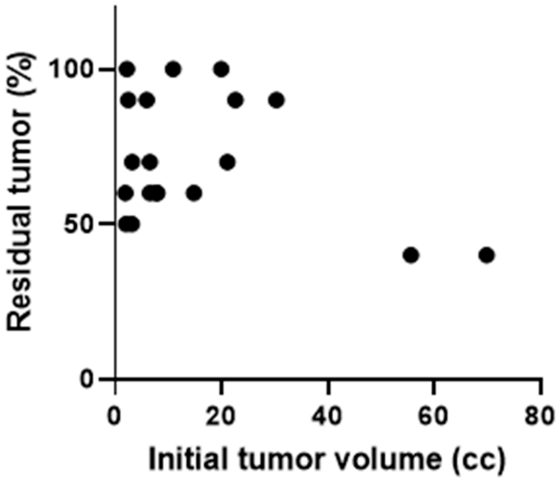

Figure S2. Relationship between initial tumor volume and volumetric response. Scatter plot showing initial internal target volume (x-axis) versus residual tumor volume at maximal response expressed as percentage of initial volume (y-axis) for all 24 patients. Larger initial tumors generally demonstrated greater absolute volume reduction, though substantial shrinkage was observed across all tumor sizes.

Table S1. Changes in Dosimetry

OAR	Mean improvement	p value
Heart Dmax (cGy)	19.0	0.0053
Bronchus Dmax (cGy)	87.4	0.0003
Spinal cord Dmax (cGy)	58.5	0.025
Esophagus Dmax (cGy)	91.3	0.0005
Lung V20 (%)	0.6	<0.0001
Lung V12.5 (%)	1.0	<0.0001
V100 (%)	3.1	<0.0001
V50 (%)	12.4	<0.0001

Table S2. Adverse Events

Category	1	2	3	4	5
Pulmonary fibrosis	1	0	0	0	0
Pneumonitis	0	1	1	0	0
Dyspnea	0	1	4	0	0
Lung infection	0	0	1	0	0
